# Supplementary figures and images for: SARS-CoV-2 spike-specific memory B cells express higher levels of T-bet and FcRL5 after non-severe COVID-19 as compared to severe disease
Source: PLoS One. 2021 Dec 22;16(12):e0261656. doi: 10.1371/journal.pone.0261656 (PMC8694470; doi:10.1371/journal.pone.0261656)

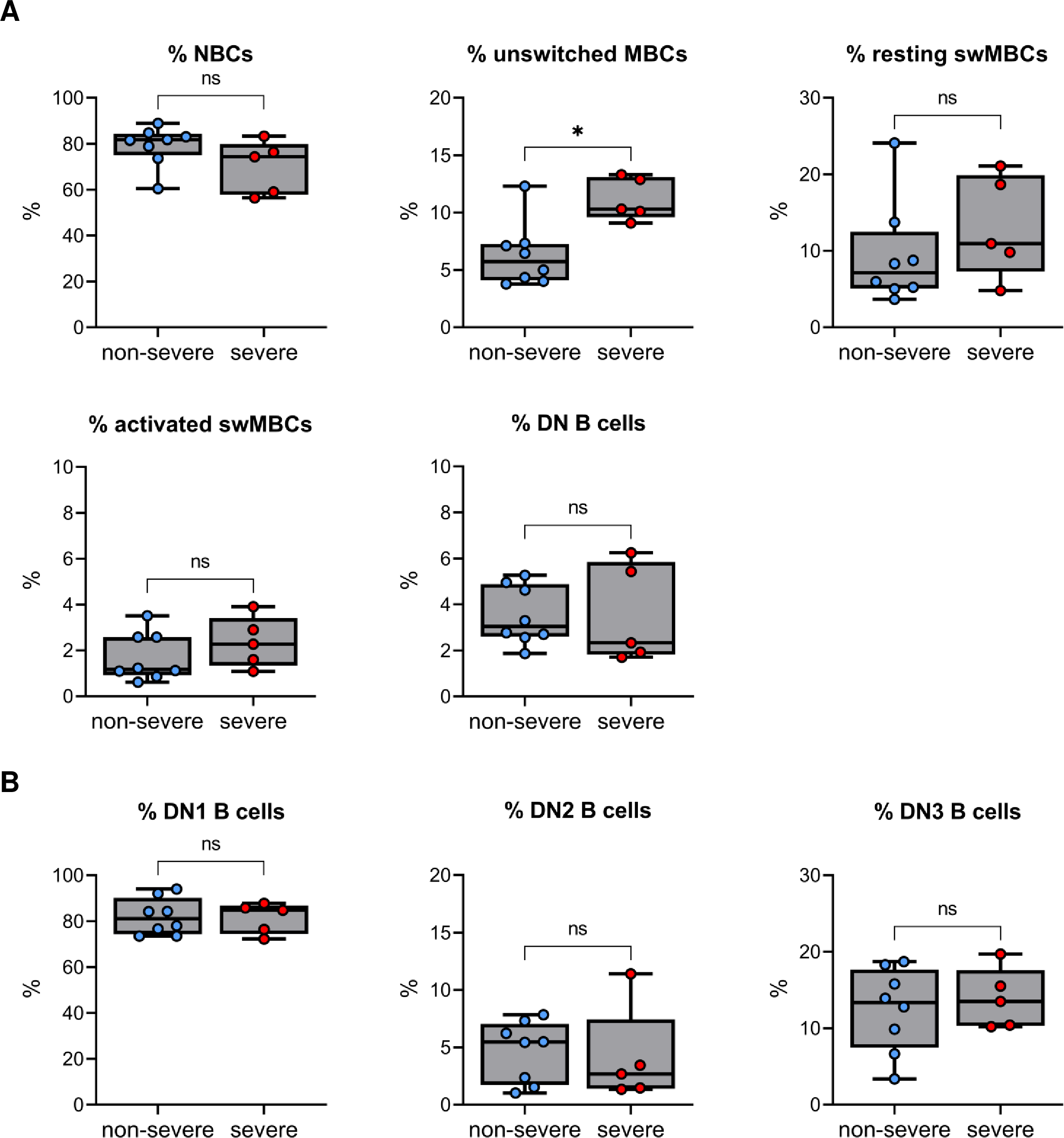

Supplement: S1 Fig — A) The percentage of naïve B cells (NBC; IgD+ CD27-), unswitched memory B cells (MBCs; IgD+ CD27+), resting switched MBCs (swMBC; IgD- CD27+CD21+), activated swMBC (IgD+ CD27+ CD21+), and double negative B cells (DN; IgD- CD27-). B) The percentage of type 1, 2, and 3 DN cells among all DN cells. Results are shown for patients who recovered from non-severe (n = 8) and severe (n = 5) COVID-19. * P < 0.05. (TIF) [file pone.0261656.s001.tif]

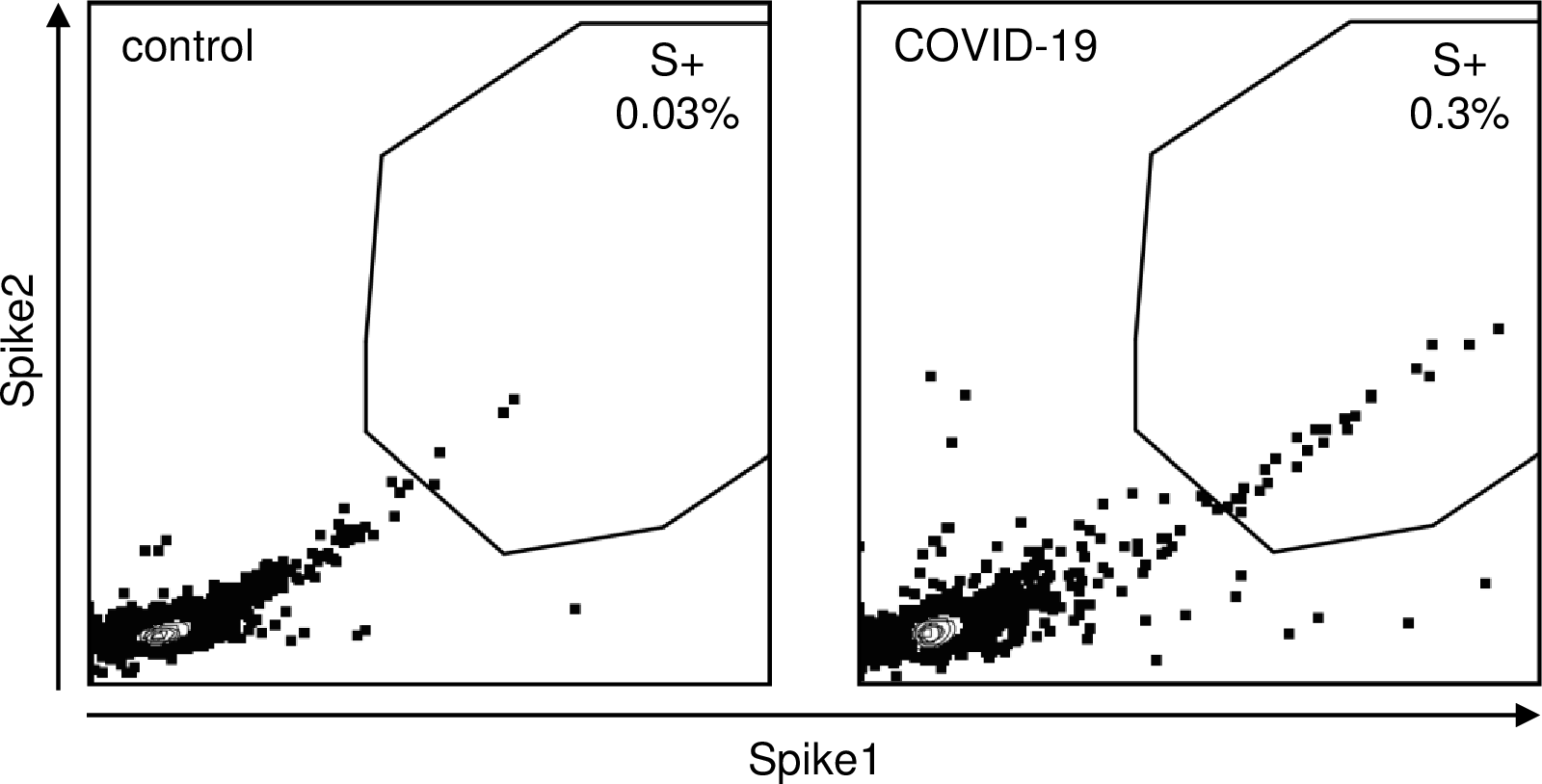

Supplement: S2 Fig — The plots show separation of spike-specific B cells from the main cluster of non-specific B cells in the bottom left corner, as well as from B cells reactive with one of the tetramers but not both. (TIF) [file pone.0261656.s002.tif]

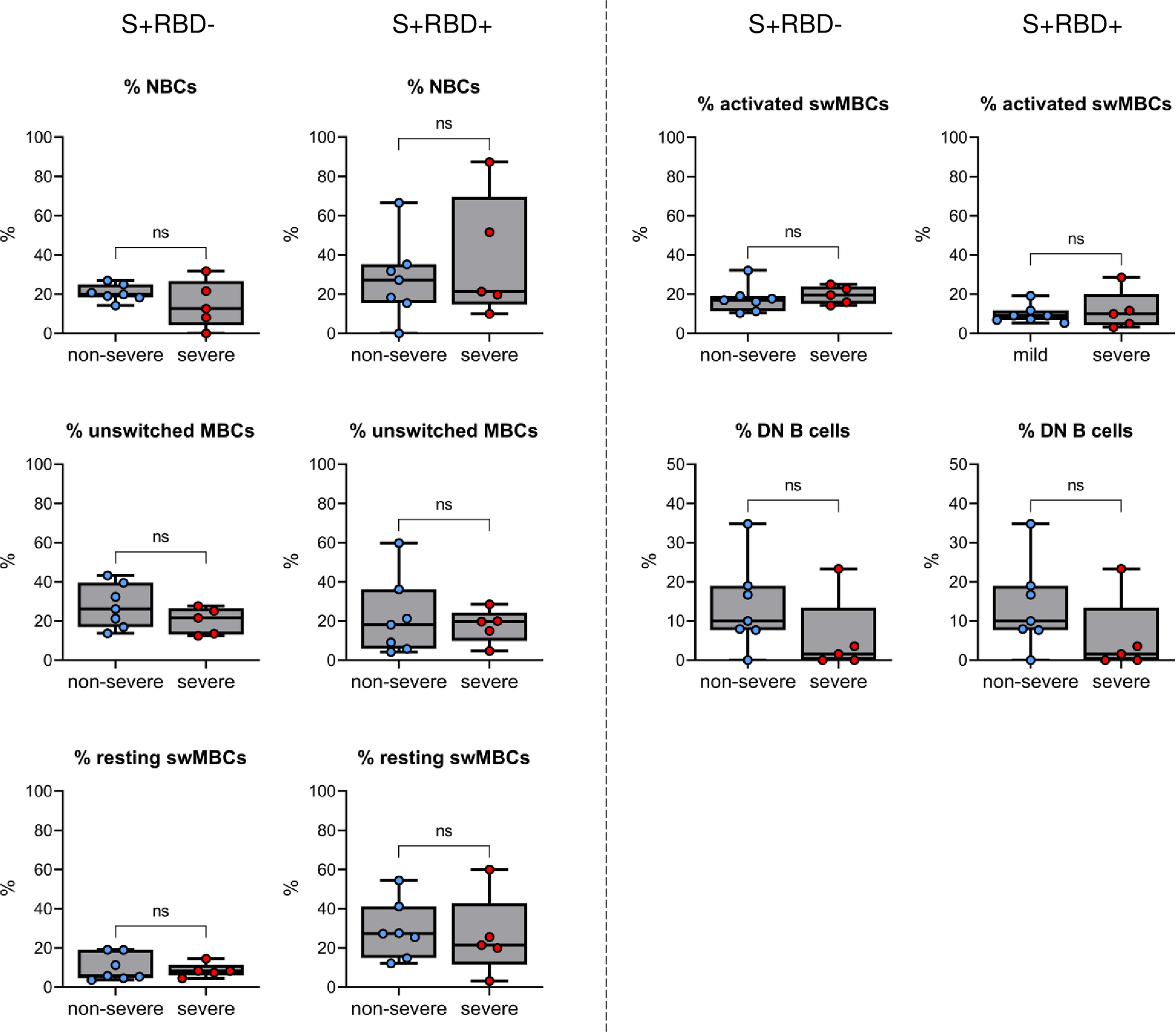

Supplement: S3 Fig — The percentage of naïve B cells (NBC; IgD+ CD27-), unswitched memory B cells (MBCs; IgD+ CD27+), resting switched MBCs (swMBC; IgD- CD27+ CD21+), activated swMBC (IgD+ CD27+ CD21-), and double negative B cells (DN; IgD- CD27-) is shown side-by-side for non-RBD-specific (S+RBD-) B cells (left) and RBD-specific (S+RBD+) B cells (right). Results are shown for patients who recovered from non-severe (n = 7) and severe (n = 5) COVID-19. (TIF) [file pone.0261656.s003.tif]

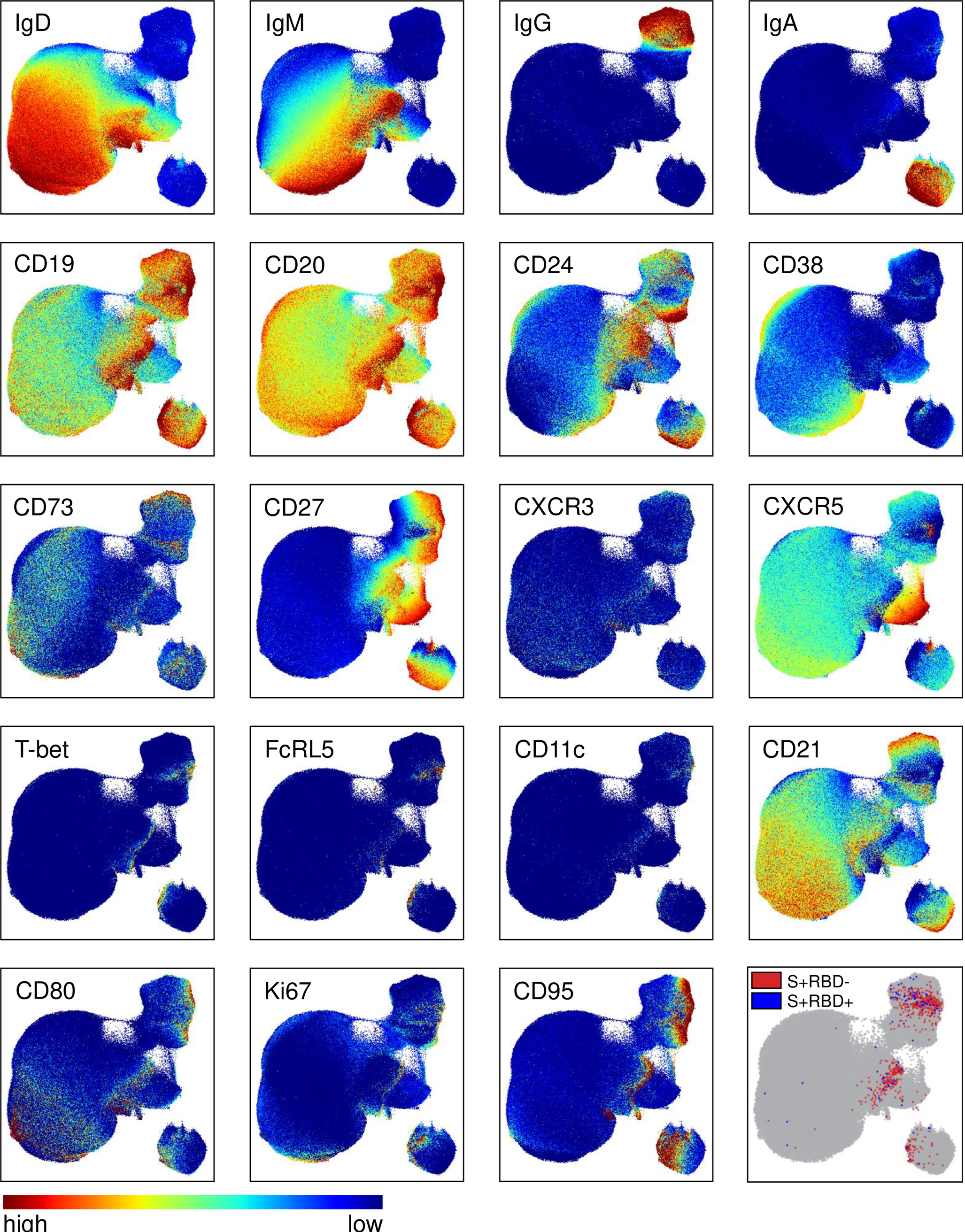

Supplement: S4 Fig — The plot in the bottom right shows the overlay of all non-RBD-specific (S+RBD-) and RBD-specific (S+RBD+) B cells onto the UMAP. (TIF) [file pone.0261656.s004.tif]

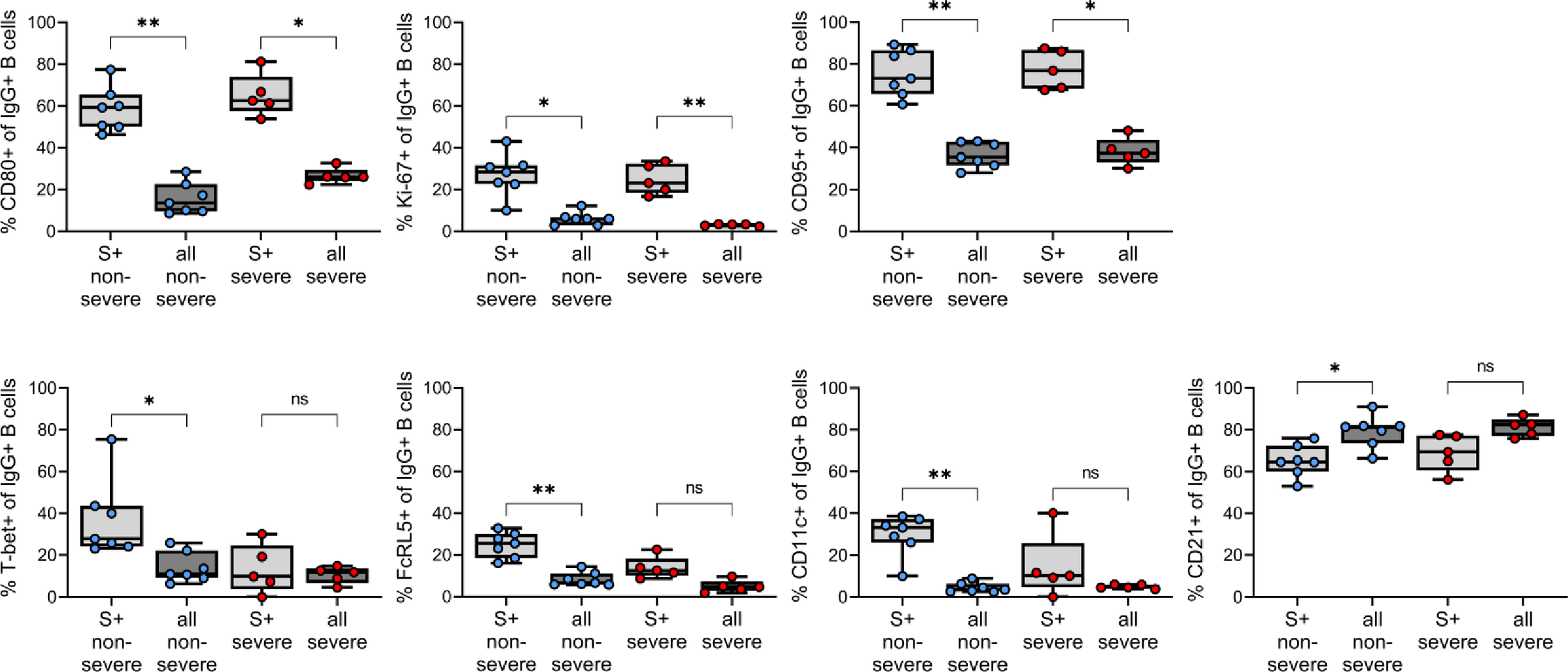

Supplement: S5 Fig — The percentage of CD80, Ki-67, and CD95 (top row) and T-bet, FcRL5, CD11c, and CD21 (bottom row) is shown for spike-specific (S+) IgG+ B cells and all IgG+ B cells in individuals who experienced non-severe (n = 7) or severe (n = 5) COVID-19. * P < 0.05; ** P < 0.01. (TIF) [file pone.0261656.s005.tif]

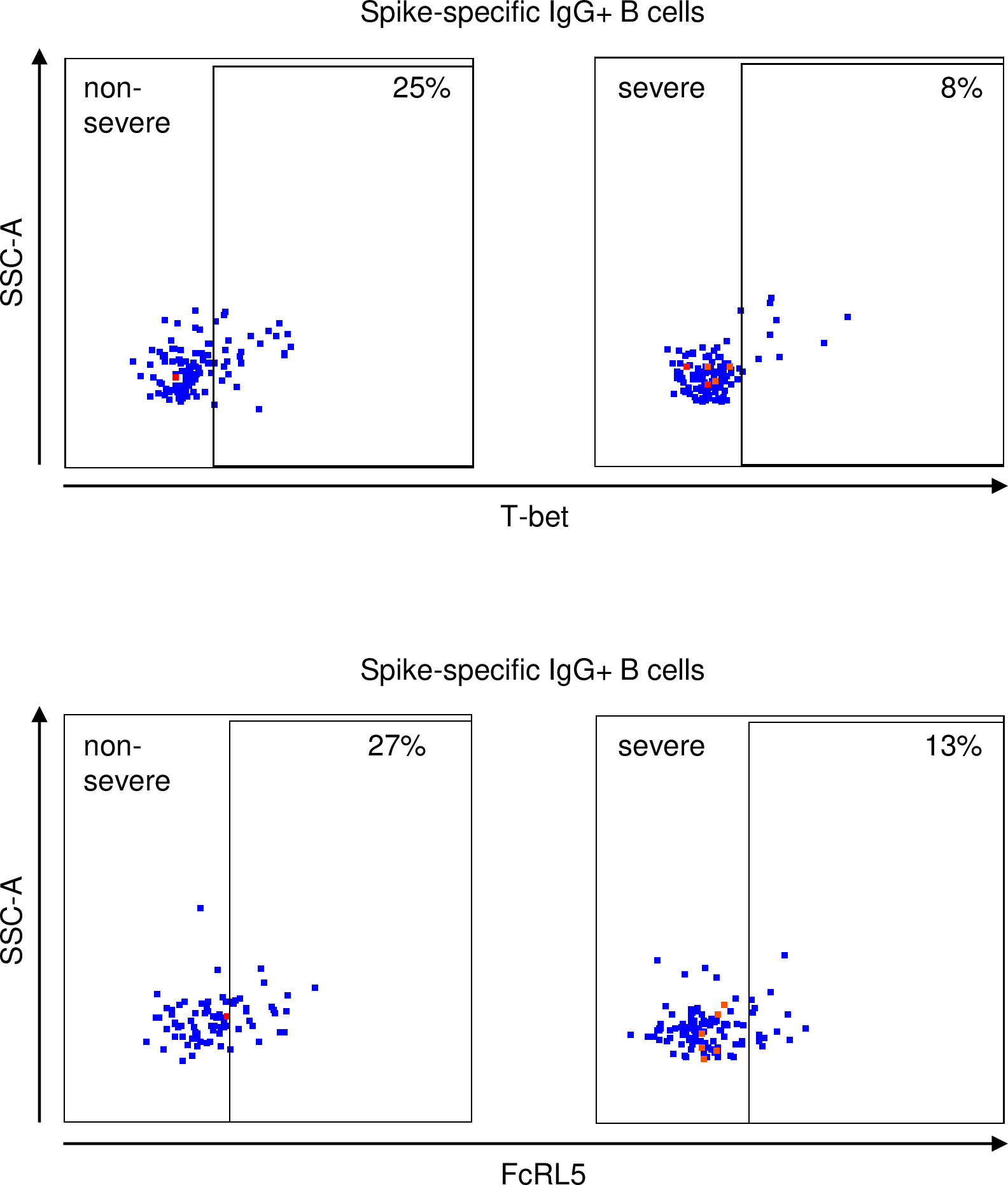

Supplement: S6 Fig — (TIF) [file pone.0261656.s006.tif]

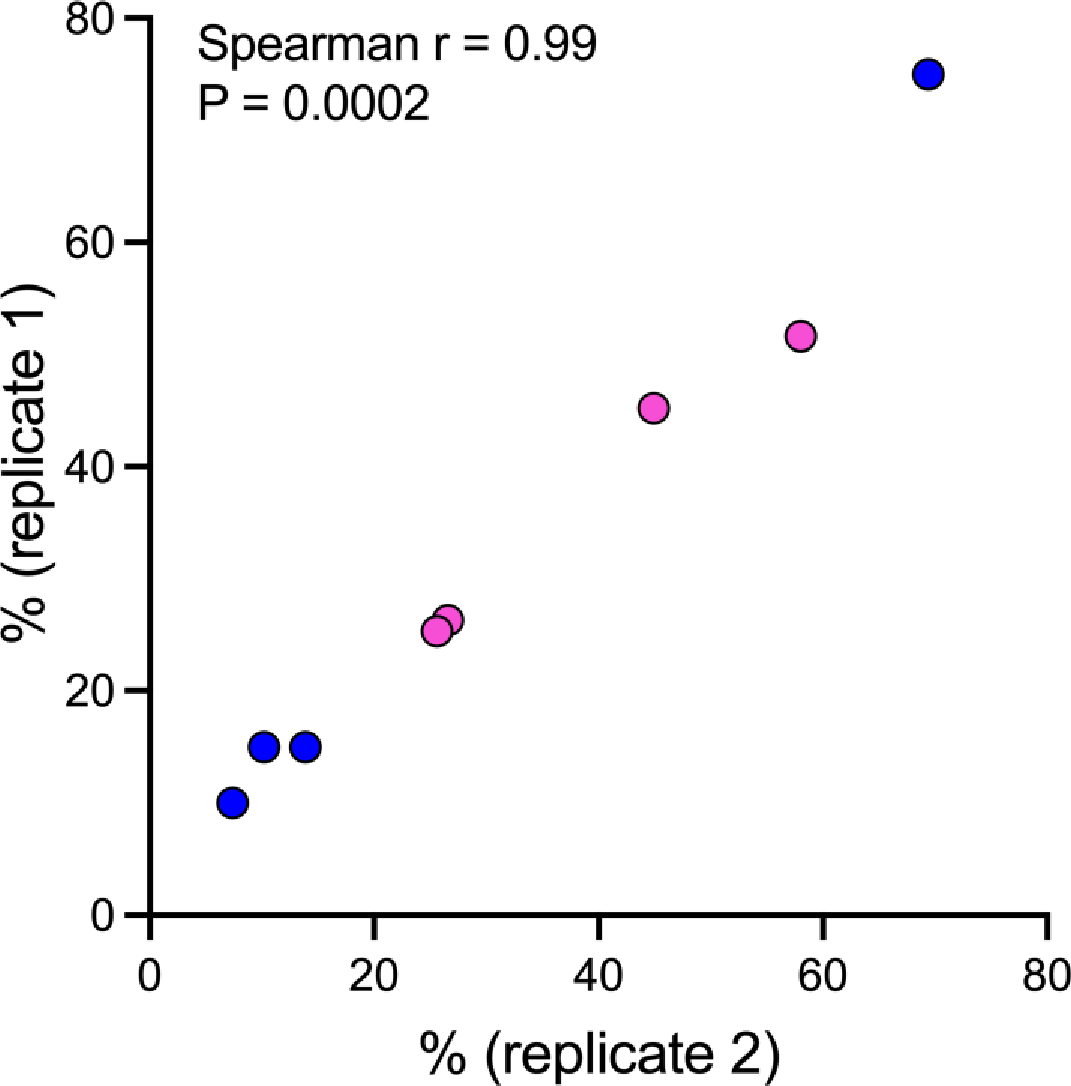

Supplement: S7 Fig — Shown are the percentages of spike-specific B cells that express T-bet, FcRL5, CD11c, and CD21 in two technical replicates, one from a non-severe case (pink) and one from a severe case (blue), that were processed and analyzed independently and blinded on separate days. Two data points (pink, ~ 25%) were overlapping and were changed slightly for visualization purposes. (TIF) [file pone.0261656.s007.tif]

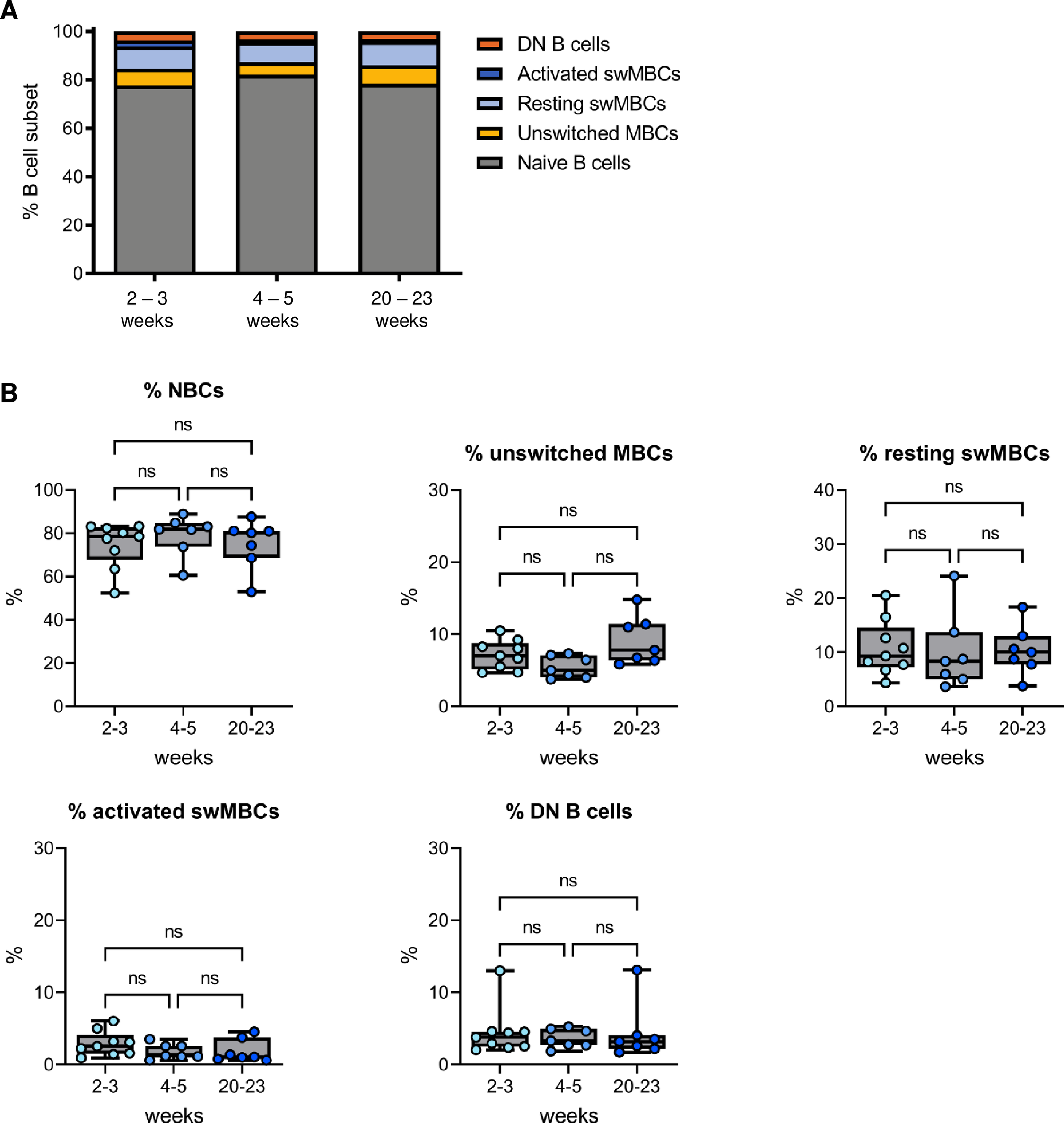

Supplement: S8 Fig — A) The median distribution of B cell subsets in recovered COVID-19 patients. B) The percentage of naïve B cells (NBC; IgD+ CD27-), unswitched memory B cells (MBCs; IgD+ CD27+), resting switched MBC (swMBCs; IgD+ CD27+ CD21+), activated swMBCs (IgD- CD27+ CD21-), and double negative B cells (DN; IgD- CD27-). In all graphs, results are shown for samples collected 2–3 (n = 9), 4–5 (n = 7), and 20–23 (n = 7) weeks post-symptom onset. (TIF) [file pone.0261656.s008.tif]

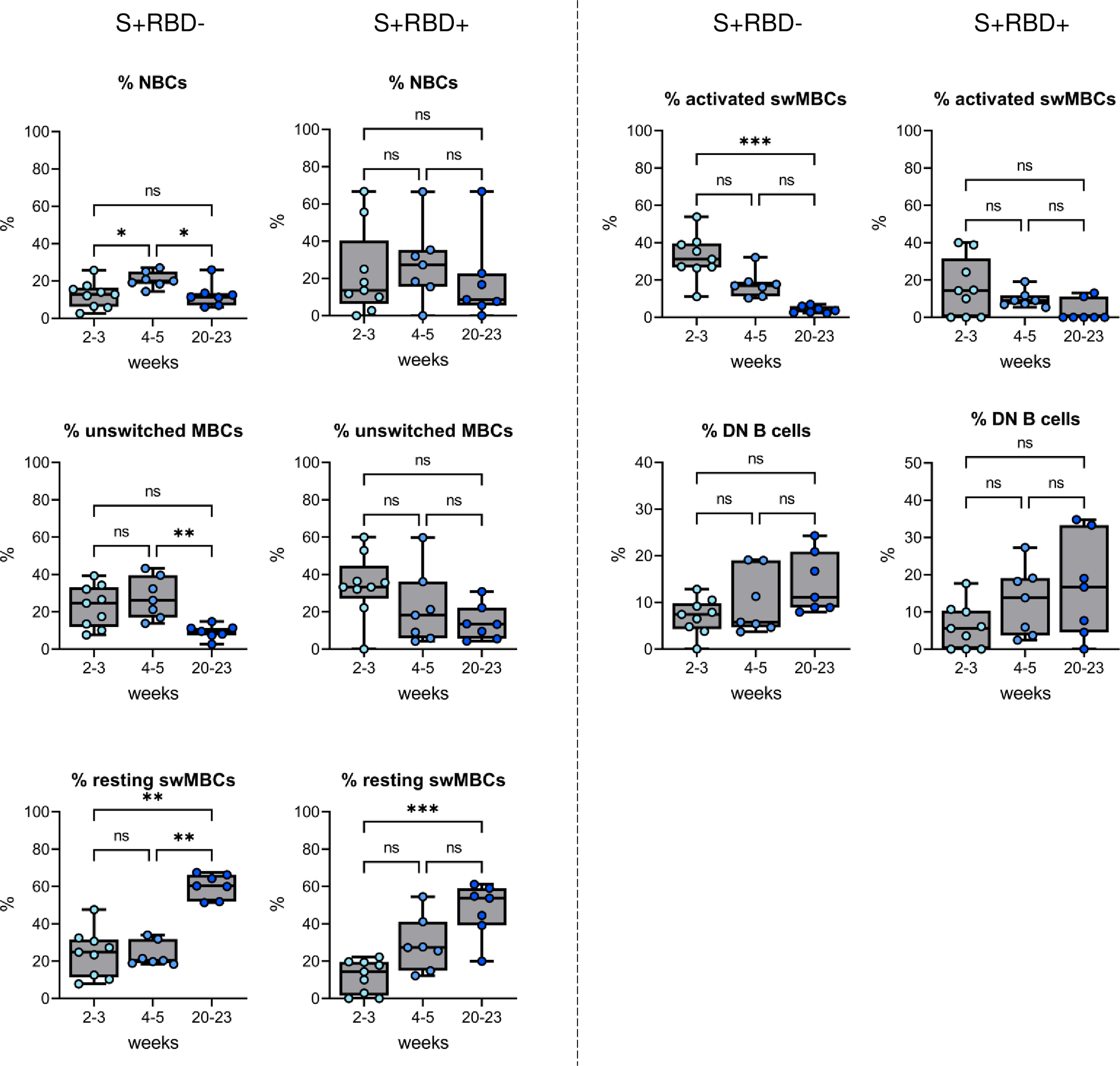

Supplement: S9 Fig — The percentage of naïve B cells (NBC; IgD+CD27-), unswitched memory B cells (MBCs; IgD+ CD27+), activated switched MBCs (swMBC; IgD- CD27+ CD21-), resting swMBC (IgD+ CD27+ CD21+), and double negative B cells (DN; IgD- CD27-) is shown side-by-side for non-RBD-specific (S+RBD-) B cells (left) and RBD-specific (S+RBD+) B cells (right). In all graphs, results are shown for samples collected 2–3 (n = 9), 4–5 (n = 7), and 20–23 (n = 7) weeks post-symptom onset. * P < 0.05; ** P < 0.01; *** P < 0.001. (TIF) [file pone.0261656.s009.tif]

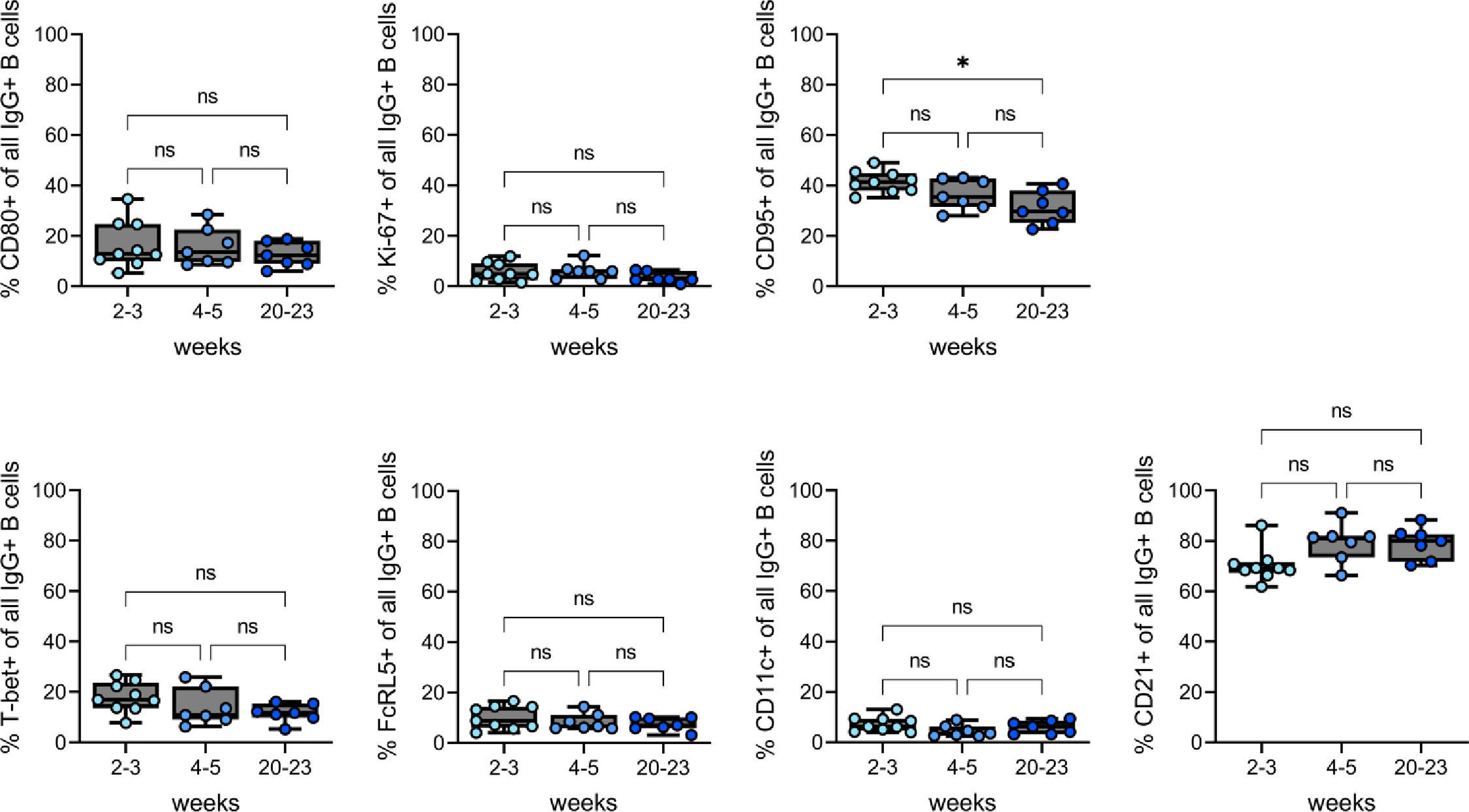

Supplement: S10 Fig — In all graphs, results are shown for samples collected 2–3 (n = 9), 4–5 (n = 7), and 20–23 (n = 7) weeks post-symptom onset. * P < 0.05. (TIF) [file pone.0261656.s010.tif]

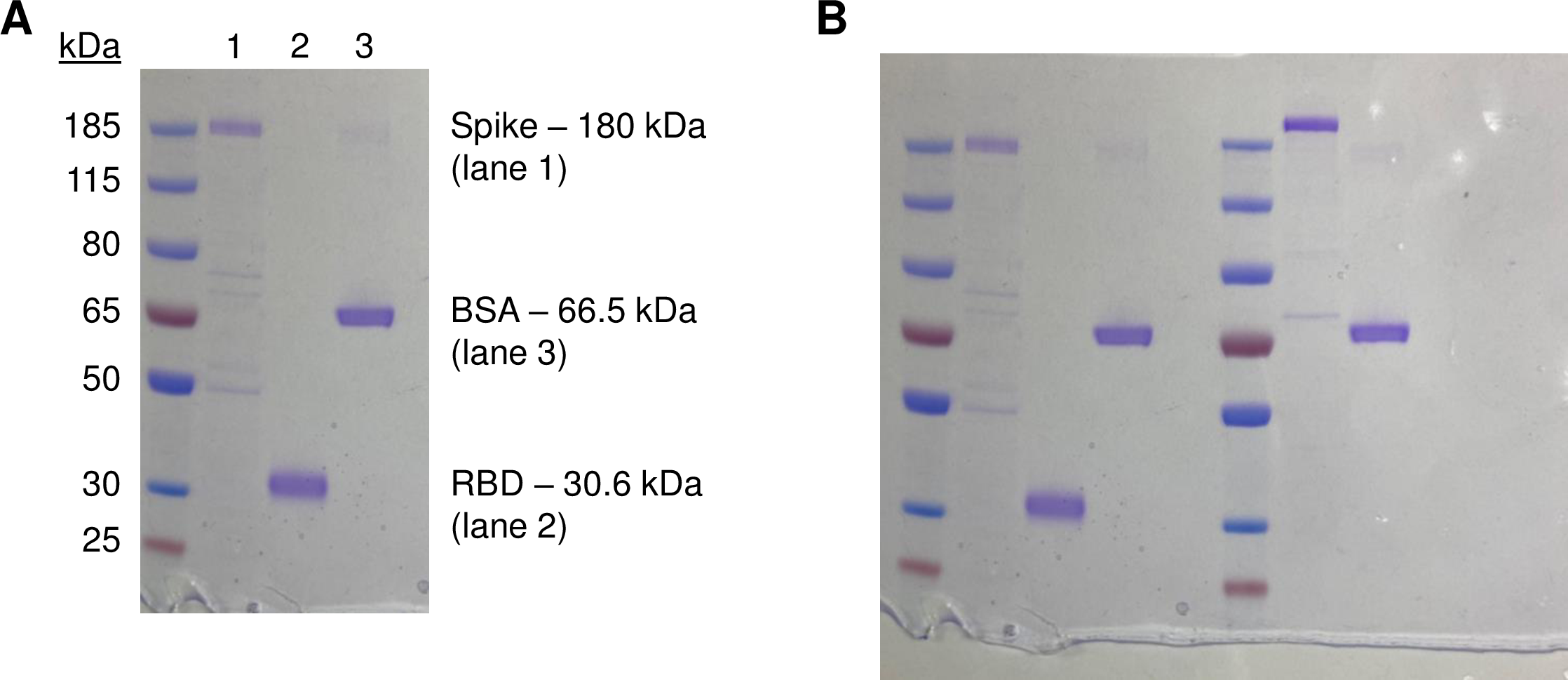

Supplement: S11 Fig — A) 800 ng of spike, RBD, and BSA was run on a 4–12% Bis-Tris gel and stained using Imperial Protein Stain. B) The full uncropped image of the gel shown in panel A. (TIF) [file pone.0261656.s011.tif]
