# Supplementary material for: SARS-CoV-2 spike-specific memory B cells express higher levels of T-bet and FcRL5 after non-severe COVID-19 as compared to severe disease
Source: PLoS One. 2021 Dec 22;16(12):e0261656. doi: 10.1371/journal.pone.0261656 (PMC8694470; doi:10.1371/journal.pone.0261656)
Supplement: S1 Table — (PDF) [file pone.0261656.s012.pdf]

Table S1: COVID-19 patient characteristics

Donor ID	Sex	Age	Race / ethnicity	Severity score	Medical history[1]	Treatment regimen[2]	Hospital stay (days)	Days PSO[3] T1	Days PSO T2	Days PSO T3
**Non-severe**										
25	M	78	White, non-Hispanic	4	DM2, OB, CKD, HTN	R + P	4	18	n.a.	156
27	F	48	White, Hispanic	4	OB, HTN	R + P	2	20	34	147
29	M	39	White, Hispanic	4	DM2, OB	R + B	2	15	29	145
32	F	59	White, non-Hispanic	4	None	R + B	2	14	31	139
34	M	59	White, Hispanic	4	OB, CRD, HTN	R + P	2	20	31	n.a.
35	M	40	Black	4	DM1, HTN	R + B	3	16	28	n.a.
38	F	68	White, Hispanic	4	DM2, OB, HTN	R + P	4	19	33	n.a.
40	F	25	White, non-Hispanic	4	Asthma	R + B	2	14	29	n.a.
33	F	20	White, Hispanic	5	OB	R + P	2	n.a.	28	138
22	M	75	White, Hispanic	5	DM2, HTN	R + B	3	17	n.a.	155
21	M	68	White, Hispanic	6	DM2, OB, HTN	R + B	9	21	n.a.	157
**Severe**										
16	M	64	White, Hispanic	7	OB	P	12	n.a.	35	n.a.
28	M	50	White, Hispanic	7	OB	R + P	22	n.a.	31	n.a.
57	F	47	White, Hispanic	7	HTN	R + B	12	n.a.	32	n.a.
66	M	36	White, Hispanic	7	OB	R + B	24	n.a.	31	n.a.
73	M	50	White, Hispanic	7	OB, HTN	R + P	11	n.a.	37	n.a.

[1] DM2, diabetes mellitus type 2; HTN, hypertension; OB, obesity; CKD, chronic kidney disease; CRD, chronic respiratory disease

[2] R, remdesivir; B, baricitinib; P, placebo

[3] PSO, post-symptom onset; n.a., not available
