# Supplementary material for: SARS-CoV-2 spike-specific memory B cells express higher levels of T-bet and FcRL5 after non-severe COVID-19 as compared to severe disease
Source: PLoS One. 2021 Dec 22;16(12):e0261656. doi: 10.1371/journal.pone.0261656 (PMC8694470; doi:10.1371/journal.pone.0261656)
Supplement: S2 Table — (PDF) [file pone.0261656.s013.pdf]

Table S2: Reagents and antibodies used for spectral flow cytometry

Reagent / antibody	Fluorophore	Clone	Company / catalog number
Live/dead stain	Zombie UV		BioLegend / 423107
Streptavidin (RBD)	BV421		BioLegend / 405226
Streptavidin (spike1)	PE		Tonbo / 504317U100
Streptavidin (spike2)	APC		Tonbo / 204317U100
CD11c	AF532	N418	Thermo / 58011482
CD19	SB645	SJ25C1	Thermo / 64019842
CD20	BV785	2H7	BioLegend / 302355
CD21	PerCP-eF710	HB5	Thermo / 46021942
CD24	BV605	ML5	BioLegend / 311123
CD27	PE-Cy7	LG.3A10	BioLegend / 124215
CD38	APC-Fire/810	HIT2	BioLegend / 303549
CD73	PerCP-Cy5.5	AD2	BioLegend / 344013
CD80	PE-Cy5	2D10	BioLegend / 305210
CD95	BUV737	DX2	BD / 612790
CXCR3	PerCP	G025H7	BioLegend / 353740
CXCR5	APC-Cy7	J252D4	BioLegend / 356925
FcRL5	BUV805	509F6	BD / 749599
IgA	FITC	IS11-8E10	Miltenyi / 130093073
IgD	PE/Dazzle	IA6-2	BioLegend / 348239
IgG	BV510	M1310G05	BioLegend / 410715
IgM	BV711	MHM-88	BioLegend / 314539
Ki-67 (intracellular)	AF700	Ki-67	BioLegend / 350529
T-bet (intracellular)	Pacific Blue	4B10	BioLegend / 644807
