# Supplementary material for: SARS-CoV-2 spike-specific memory B cells express higher levels of T-bet and FcRL5 after non-severe COVID-19 as compared to severe disease
Source: PLoS One. 2021 Dec 22;16(12):e0261656. doi: 10.1371/journal.pone.0261656 (PMC8694470; doi:10.1371/journal.pone.0261656)
Supplement: Attachment — Submitted filename: Response_to_Reviewers.pdf [file pone.0261656.s014.pdf]

We would like to thank the reviewers for their feedback. Please find below a point-by-point reply to reviewer comments. Changes in the manuscript are highlighted in red text.

Part I – Summary

Please use this section to discuss strengths/weaknesses of study, novelty/significance, general execution and scholarship.

Reviewer #1: In this manuscript by Reyes et al., the authors examine the B cell response following severe and non-severe SARS-CoV-2 infection using spectral flow cytometry. The authors identify spike-specific memory B cells and assess the expression of markers that define memory B cell subsets across multiple time points. They find that memory B cells present at 5 months post infection display reduced expression of T-bet, Fcrl5, and CD11c and increased expression of CD21. These findings are consistent with published work showing that SARS-CoV-2 infection induces a durable memory B cell response.

Memory B cells are a critical component of long-term protective immunity. Understanding the dynamics of the memory B cell response following SAR-CoV-2 infection is important in predicting how these cells will response upon antigen re-encounter. Similarly, elucidating how the severity of infection influences the composition of the memory B cell response is necessary to predict the duration and quality of protective immunity elicited by infection. Therefore, this study contributes to an important growing set of studies assessing the memory B cell response following SARS-CoV-2 infection or vaccination. However, technical limitations associated with the study and some conclusions that are not fully supported by the data limit the impact of this study.

Please see our response to the reviewer's more detailed comments below.

Reviewer #2: In this study, Reyes et al. analyzed phenotypes of SARS-CoV-2 Spike specific memory B cells in 8 individuals with "non-severe" disease and 5 individuals with "severe" disease. They observed that S-specific B cells from patients with milder disease showed increased expression of T-bet, FcRL5, and CD11c. Although the experiments in the study were performed well, and clearly described, I don't really see any new findings of significance here. Methods and findings in this study are very similar to those of a paper published more than six month ago by Ogega, et al. (Ogega, J Clin Invest. 2021 Apr 1). Both studies compared B cell phenotypes between convalescent individuals with mild or severe COVID-19 disease, and both studies found increased expression of FCRL5 on RBD-specific B cells after mild disease. In addition, multiple papers have now been published that quantitated and analyzed S-specific B cells up to a year after infection, providing stronger evidence of the durability of B cell responses than the phenotyping in this study. Thus, I find the observations in this study to be incremental and of limited significance.

We agree with the reviewer that many of our findings are similar to those published by others. However, our study includes additional information that has not been reported by others, including the observation that more spike-specific B cells express T-bet after non-severe COVID-19 than severe COVID-19, a detailed phenotyping of these T-bet$^+$ B cells, and the dynamics of T-bet$^+$ B cells in the weeks and months after infection. While it is true that several papers have reported on S-specific B cell responses up to a year after infection, we are not aware of any studies that have directly compared the level of immunological protection between individuals who suffered from non-severe and severe disease, up to a year after infection. We therefore believe that the results of our phenotyping study will

be of interest to B cell immunologists in general and those studying immune responses to viral infections in particular, and may spur further comparison between the two groups over time.

Part II – Major Issues: Key Experiments Required for Acceptance

Please use this section to detail the key new experiments or modifications of existing experiments that should be absolutely required to validate study conclusions.

Generally, there should be no more than 3 such required experiments or major modifications for a "Major Revision" recommendation. If more than 3 experiments are necessary to validate the study conclusions, then you are encouraged to recommend "Reject".

Reviewer #1: 1) The authors conclude that the memory B cell response elicited during non-severe SARS-CoV-2 infection may be of higher quality than the response after severe disease. However, the authors show that the memory B cell response in severely and non-severely infected individuals is comparable in similar in magnitude and phenotype. The authors observe no significant difference in CD80, Fcrl5, CD11c, Cd21, Ki67, or CD95 between the groups. The only significant difference observed between the groups is a decrease in T-bet expression in IgG+ spike-specific B cells following severe infection, which appears driven by high expression in 3/8 samples. The authors also do not identify a significant difference in the phenotype of T-bet+ IgG+ B cells.

Therefore, the data does not support the conclusion in the title that memory B cells induced following severe infection do not express makers of durable immunity. The authors should revise the title and text to accurately reflect their data that memory B cells induced following non-severe and severe infection are phenotypically similar. Alternatively, the author should expand their study to increase the number of individuals in the severe and non-severe groups to determine if the trend toward decreased Fcrl5 and CD11c reaches statistical significance.

Our data indeed shows that the memory B cells response after non-severe and severe COVID-19 is similar in magnitude. However, we do not agree with the reviewer that the phenotype of the B cell response is similar. We observed higher expression of T-bet, FcRL5, and CD11c, and a decrease in CD21 expression in spike-specific IgG^{+} B cells after non-severe COVID-19 as compared to the total IgG^{+} B cell population. None of these markers were upregulated after non-severe COVID-19 in spike-specific IgG^{+} B cells as compared to the total B cell population. When tested directly between the non-severe and severe groups, we observed increased expression in T-bet and FcRL5 in the non-severe group as compared to the severe group. We have updated the analysis presented in Figure 4 to reflect this, and have moved the analysis of marker expression between spike-specific IgG^{+} B cells and all IgG^{+} B cells to the new Figure S5. We have made several changes throughout the manuscript to more carefully describe these results, including the addition of a sentence in the abstract stating that the phenotype of T-bet^{+} spike-specific B cells did not differ between the two groups.

2) Related to the above point, the strength of many of the conclusions made in this paper are limited by small group size. The authors acknowledge this limitation and suggest that the similarity between their results and other published work with larger cohorts gives them confidence in their results. However, the small group size in the severe versus non-severe infected individuals and the low number of spike-

specific B cell make it difficult to have confidence in the conclusions of this study.

This study was designed with carefully matched sample collection at 4 – 5 weeks post-symptom onset in individuals who recovered from non-severe and severe disease to account for rapid changes in the abundance of activated B cell subsets over time. Due to the strict inclusion criteria of the study, we unfortunately were unable to include additional samples in this study. Given that our findings are in line with those reported by others (most notably Ogega *et al.*, who also provided evidence for increased expression of FcRL5 after non-severe disease as compared to severe disease), we continue to have confidence in our observations.

Reviewer #2: (No Response)

Part III – Minor Issues: Editorial and Data Presentation Modifications

Please use this section for editorial suggestions as well as relatively minor modifications of existing data that would enhance clarity.

Reviewer #1: 1) The spike gating show in Fig. 3a is not convincing as many of the cells that are labeled S+ do not show clear separation. The authors should validate that the Spike+ cells identified in their analysis response upon restimulation with spike protein.

We have carefully gated spike-specific B cells, as shown more clearly in the zoomed in plots below. The plots show separation of spike-specific B cells from the main cluster of non-specific B cells in the bottom left corner, as well as from B cells reactive with one of the tetramers but not both. This plot has been included as Figure S2 in the revised version of the manuscript.

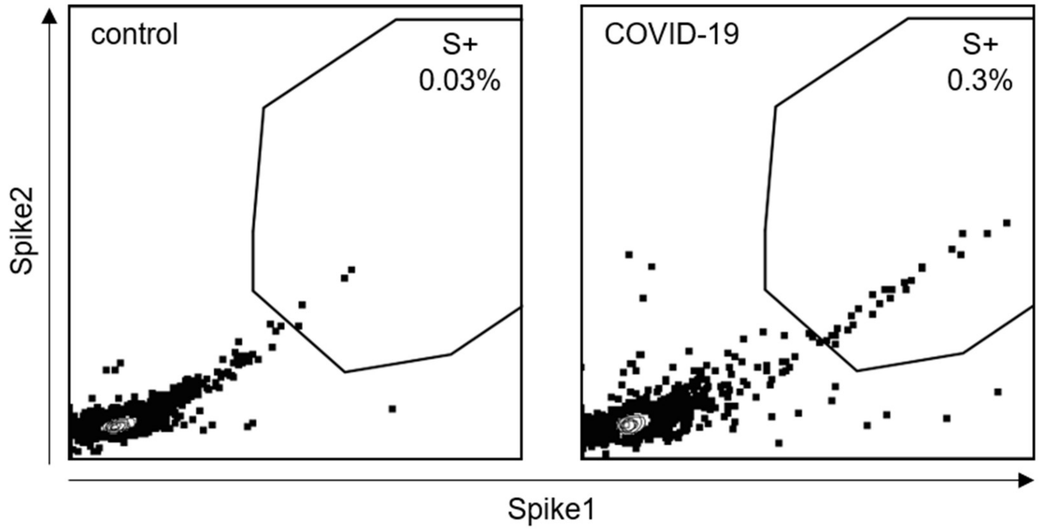

2) Representative flow cytometry plots should be shown to assess the robustness of the differences seen in the percentage of T-bet+ IgG+ spike specific B cells.

We have included the representative flow cytometry plots of the percentage of T-bet$^+$ and FcRL5$^+$ spike-specific IgG$^+$ B cells shown below as Figure S6 of the revised manuscript.

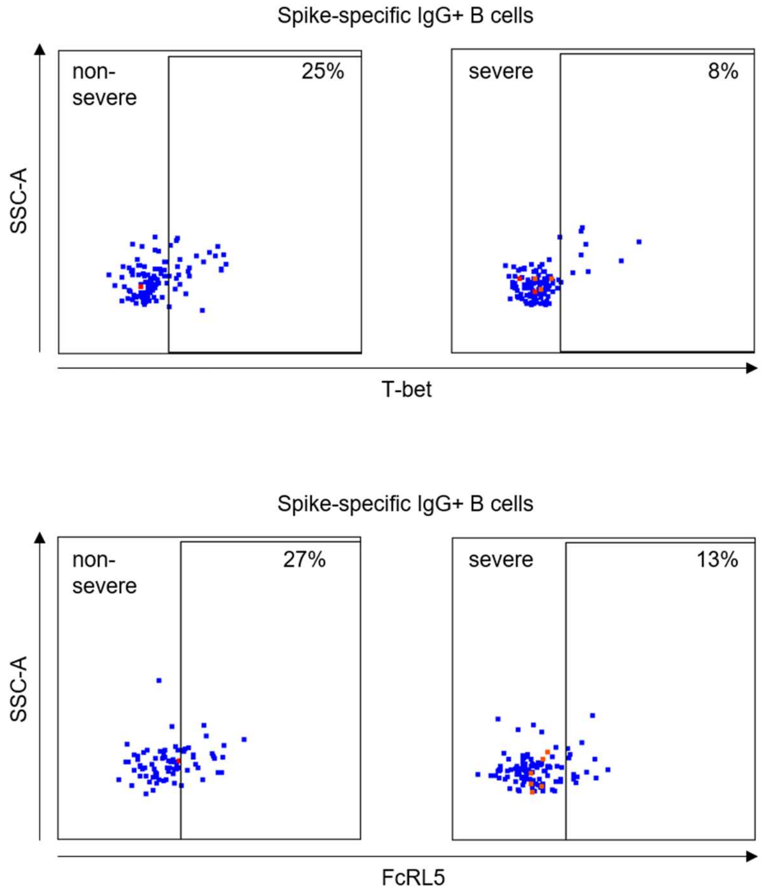

Reviewer #2: (No Response)
